# Mutagenesis analysis of the zinc-finger antiviral protein

**DOI:** 10.1186/1742-4690-7-19

**Published:** 2010-03-13

**Authors:** Xinlu Wang, Fengxiang Lv, Guangxia Gao

**Affiliations:** 1 Key Laboratory of Infection and Immunity, Institute of Biophysics, Chinese Academy of Sciences, Beijing 100101, China

## Abstract

**Background:**

The zinc-finger antiviral protein (ZAP) specifically inhibits the replication of certain viruses, including murine leukemia virus (MLV), by preventing the accumulation of viral mRNA in the cytoplasm. ZAP directly binds to the viral mRNA through the zinc-finger motifs and recruits the RNA exosome to degrade the target RNA. RNA helicase p72 is required for the optimal function of ZAP. In an attempt to understand the structure-function relationship of ZAP, we performed alanine scanning analysis.

**Results:**

A series of ZAP mutants was generated, in which three consecutive amino acids were replaced with three alanines. The mutants were analyzed for their antiviral activities against pseudotyped MLV vector. Out of the nineteen mutants analyzed, seven displayed significantly lower antiviral activities. Two mutations were in the very N-terminal domain, and five mutations were within or around the first and second zinc-finger motifs. These mutants were further analyzed for their abilities to bind to the target RNA, the exosome, and the RNA helicase p72. Mutants Nm3 and Nm63 lost the ability to bind to RNA. Mutants Nm 63 and Nm93 displayed compromised interaction with p72, while the binding of Nm133 to p72 was very modest. The interactions of all the mutants with the exosome were comparable to wild type ZAP.

**Conclusions:**

The integrity of the very N-terminal domain and the first and second zinc-finger motifs appear to be required for ZAP's antiviral activity. Analyses of the mutants for their abilities to interact with the target RNA and RNA helicase p72 confirmed our previous results. The mutants that bind normally to the target RNA, the exosome, and the RNA helicase p72 may be useful tools for further understanding the mechanism underlying ZAP's antiviral activity.

## Background

Host restriction factors inhibit retrovirus infection at different steps in the retroviral life cycle by various mechanisms [1-3]. The zinc-finger antiviral protein (ZAP) was originally recovered from a screen for genes conferring resistance by cells to infection by Moloney murine leukemia virus (MLV) [4]. In addition to MLV, ZAP was later found to inhibit the replication of Ebola virus (EBOV) and Marburg virus (MARV) [5], and multiple members of alphaviruses, including Sindbis virus (SINV) [6]. The expression of ZAP does not induce a broad-spectrum antiviral state, as the replication of some viruses, including herpes simplex virus type 1 and yellow fever virus, is not affected in ZAP-expressing cells [6].

Analysis of the step at which ZAP inhibits MLV infection revealed that the formation and nuclear entry of the viral DNA were normal, but the viral mRNA level was significantly reduced in the cytoplasm of ZAP-expressing cells. The half-lives of the viral mRNA in the cytoplasm were about 2.5 h and 0.5 h in the control and ZAP-expressing cells, respectively, indicating that ZAP promotes the degradation of viral mRNA in the cytoplasm [4,7].

ZAP directly binds to the target RNA and recruits the RNA processing exosome, a 3'-5' exoribonucleases complex consisting of at least nine components [7,8], to degrade the RNA. The rat ZAP recruits the exosome through direct binding to the exosome component Rrp46. The RNA helicase p72 directly interacts with ZAP and is required for optimal function of ZAP [9]. The sensitivity of certain viruses to the inhibitory effect of ZAP seems to be determined by the presence of the ZAP responsive element (ZRE) in the viral mRNA. The ZRE in MLV was mapped to the 3' long terminal repeat (LTR), while multiple fragments of SINV are responsive to ZAP [10]. The sensitive sequences in EBOV and MARV were mapped to the L fragment [5]. Among these ZREs, no obvious conserved motifs or secondary structures predicted using currently available softwares have been observed. The only common feature is that the minimum length of these ZREs is about 500 nucleotides.

In the N-terminal domain of ZAP, there are four CCCH-type zinc-finger motifs. Disruption of the second or fourth finger abolished the antiviral activity of ZAP, while disruption of the first or third finger had little effect [10]. When the N-terminal domain of the 254 amino acids of ZAP is fused to the zeocin resistance gene (NZAP-Zeo), the fusion protein displays the same antiviral activity as the full-length protein [4], suggesting that the N-terminal domain is the major functional domain. Indeed, the interacting regions of ZAP with the target RNA, the exosome, and the RNA helicase p72 were all mapped to this domain [7,9,10].

As a step to further understanding how ZAP organizes the RNA degradation machinery to degrade viral RNA, we used the alanine scanning method to explore the structure-function relationship of the N-terminal domain of ZAP.

## Results

### Antiviral activity of the ZAP mutants

A series of NZAP-Zeo mutants, in which three consecutive amino-acids were substituted with three alanines, was constructed and packaged into MLV vector to transduce Rat2 cells. The transduced cells were selected in zeocin-containing medium and pooled for further analyses. Out of the 25 mutants constructed, 19 mutants rendered cells resistant to zeocin selection. However, the remaining 6 mutants failed to do so. To rule out the possibility that the mutations affected the packaging into MLV vector, these 6 constructs were also stably transfected into Rat2 cells, but none of the cells survived the selection. Why these mutants failed to render the cells resistant to zeocin remains elusive. One possibility is that the mutations interfered with the function of the zeocin resistance gene. Another possibility is that the mutations interfered with the folding of the protein, resulting in very low levels of expression.

**Figure 1:**
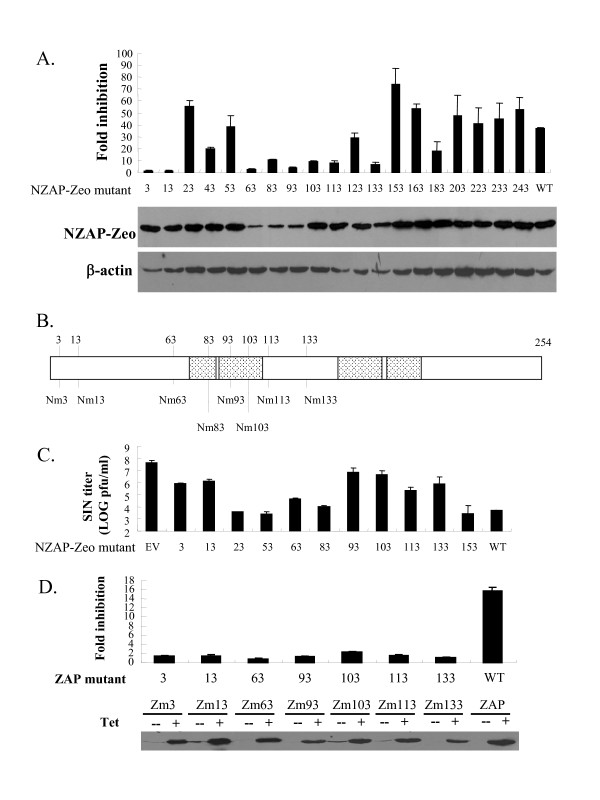
**Antiviral activity of the ZAP mutants**. (A) The Rat 2 cells expressing the indicated NZAP-Zeo-myc mutants were challenged with MLV-luc. At 48 h post infection the cells were lysed, and luciferase activity was measured. Fold inhibition was calculated as the luciferase activity in the control cells divided by that in the NZAP-Zeo-myc expressing cells (upper panel). The expression of the mutant proteins was analyzed by Western blotting (lower panel). The fold inhibition data are mean + SD of three independent experiments. (B) Schematic representation of the mutation positions in the mutants with reduced antiviral activity. The zinc-finger domains are represented as shaded boxes. (C) The Rat2 cells expressing the indicated NZAP-Zeo-myc mutants were infected with SINV for 1 h. At 48 h post infection, the supernatants were collected and the virus was titrated. EV: empty vector-transduced cells; WT: wild-type NZAP-Zeo transduced cells. (D) 293TRex cells stably expressing the ZAP mutants in a tetracycline-inducible manner were infected with MLV-luc. At 6 h post infection, the cells were equally divided into two dishes, with one mock treated and the other treated with tetracycline. At 48 h post infection the cells were lysed and luciferase activity was measured. Fold inhibition was calculated as the luciferase activity in the mock-treated control cells divided by that in the ZAP-expressing cells (upper panel). The tetracycline induced protein expression was confirmed by Western blotting (lower panel). The fold inhibition data are mean + SD of three independent experiments.

The expression levels of the zeocin resistant mutants were measured by Western blotting. The mutants were expressed at comparable levels except for NZAP-Zeo mutants 63, 83 and 93, which were expressed at lower levels than the rest (Fig. 1A, lower panel). To assess the antiviral activity of the ZAP mutants, the cells were challenged with VSVG-pseudotyped MLV-luc, and the activity was measured and presented as fold inhibition. Eight mutants (NZAP-Zeo mutants 3, 13, 63, 83, 93, 103, 113 and 133) displayed significantly reduced activity compared with the wild-type ZAP (Fig. 1A, upper panel). The positions of the eight mutants whose activities were significantly reduced are summarized (Fig. 1B). Five of them are within or around the first and second zinc-finger motifs. Out of the other three, two (Nm3 and Nm13) are localized at the very N-terminal end of the protein.

Out of the eight mutants that displayed significantly reduced antiviral activity, seven (Nm3, 13, 63, 93, 103, 113 and 133) were further pursued. The reason that mutant Nm83 was not included was that it was expressed at a relatively low level but displayed much higher activity than Nm 63 and Nm 93, which were expressed at comparable levels. To assess whether the mutations specifically affected the antiviral activity of ZAP against MLV, the mutants with reduced activity against MLV were also assayed for their activity to inhibit the propagation of SINV. As expected, the inhibitory effect against SINV of the seven mutants was significantly impaired (Fig. 1C). In contrast, the mutants (Nm23, 53 and 153) whose antiviral activity against MLV was not affected were also active against SINV (Fig. 1C). To confirm that the mutations affected the full-length ZAP protein as NZAP-Zeo, the seven mutations were also introduced into the full-length ZAP. The proteins were expressed in 293TRex cells in a tetracycline-inducible manner and assayed for their antiviral activities against MLV-luc. As expected, all these mutants displayed very low antiviral activity compared with the wild-type ZAP (Fig. 1D).

### Activities that bind the target RNA

**Figure 2:**
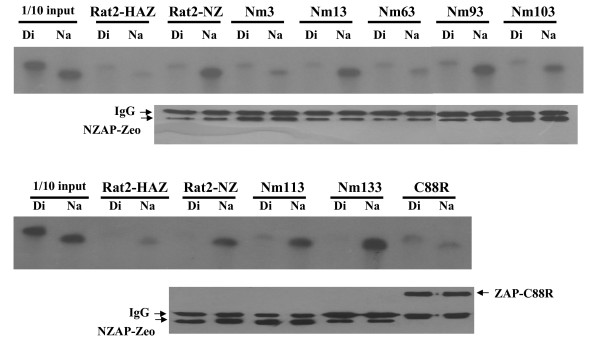
**The activity of the ZAP mutants to bind the target RNA**. The lysates of Rat2 cells expressing the indicated NZAP-Zeo-myc mutants were mixed with 9E10 anti-Myc antibody and proteinG-agarose resin for 2 h to immobilize ZAP to the resin. The resins were washed and incubated with the indicated 32-P labeled. RNA probes for 30 minutes in binding buffer and then washed three times with the binding buffer. Bound RNAs were eluted by boiling in RNA sample buffer, subjected to urea-polyacrylamide gel electrophoresis, and detected by autoradiography. Bound ZAP proteins were eluted by boiling in protein sample buffer and detected by Western blotting. Rat2-HAZ: Rat2 cells transduced with empty vector; Rat2-NZ: Rat2 cells expressing wild-type NZAP-Zeo-myc; Nm: Rat2 cells expressing NZAP-Zeo-myc mutants; C88R: cells expressing full-length ZAP-C88R-myc mutant as a negative control.

It has been previously reported that ZAP directly binds to ZRE-containing RNA [10]. To understand how the antiviral activity of the mutants was affected, we first measured the ability of these mutants to bind to the target RNA by *in vitro *binding assay. The ZRE-containing RNA Na, which has been reported to bind to ZAP [10], was used for the assay. The non-ZRE-containing RNA Di, which failed to bind to ZAP [10], was used as a negative control for non-specific binding. The NZAP-Zeo-myc mutants were immobilized on the beads and incubated with the RNAs. The bound RNA was detected by autoradiography following electrophoresis. As shown in Figure 2, the binding of Nm3, Nm63 to the target RNA was significantly reduced, while the binding of mutants Nm 13, Nm 93, Nm 113 and Nm 133 was comparable to the wild-type NZAP-Zeo. The mutant Nm103 displayed moderate binding to the target RNA. Western blotting results indicated that comparable amounts of the proteins were immobilized on the beads (Fig. 2, lower panel).

### Interaction with the RNA exosome

**Figure 3:**
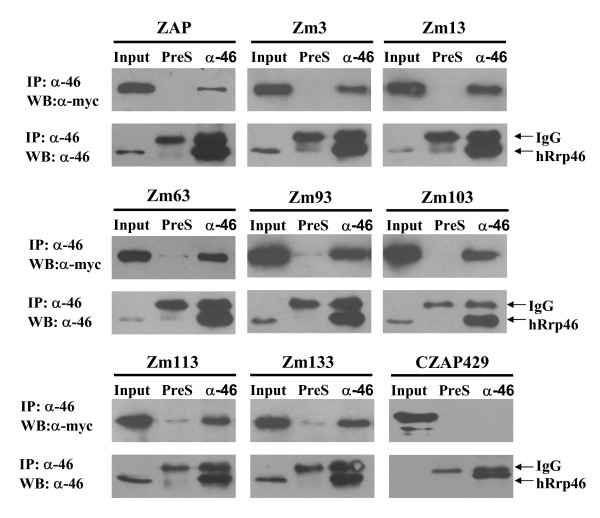
**Interactions of the ZAP mutants with the RNA exosome**. 293TRex-ZAP mutant cells were treated with tetracycline to induce ZAP expression. CZAP429-myc was expressed by transient transfection into HEK 293T cells. The cells were lysed in the lysis buffer in the presence of 100 μg/ml RNase A. The proteins were immunoprecipitated with rabbit anti-hRrp46p (α-46) or pre-immune serum (PreS) and Western blotted with the anti-myc antibody (upper panel) or anti-hRrp46 antibody (lower panel). Input: total cell lysate.

The RNA processing exosome is an evolutionarily highly conserved 3'-5' exoribonucleases complex existing in both the nucleus and the cytoplasm [11-13]. The cytoplasmic exosome plays a key role in the degradation of aberrant or unused intermediate mRNAs and ARE containing mRNAs [14-17]. ZAP recruits the exosome to degrade the target RNA through directly binding to the exosome component [7]. To examine whether the mutations affected the interaction between ZAP and the exosome, co-immunoprecipitation assays were performed. The myc-tagged ZAP mutants were expressed in 293TRex cells and analyzed for their interaction with the endogenous exosome component Rrp46. To prevent non-specific RNA tethering, RNase A was added to the cell lysis buffer. Immunoprecipitation of the endogenous exosome coprecipitated all the ZAP mutants, but not a truncated form of ZAP (Fig. 3), suggesting that the mutations did not affect the binding of these mutants to the exosome.

### Interaction with p72 RNA helicase

**Figure 4:**
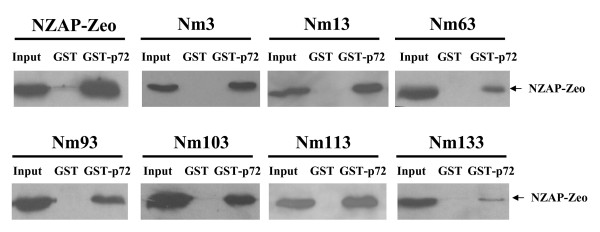
**Interactions of the ZAP mutants with the RNA helicase p72**. Bacterially expressed GST or GST-p72 was immobilized onto glutathione-Sepharose 4B resin. The resins were washed and incubated with cell lysates of the NZAP-Zeo-myc mutants in the presence of RNase A for 2 h. The resins were washed and boiled in the sample loading buffer. The proteins were resolved by SDS-PAGE and detected by Western blotting using the anti-myc antibody. Input: total cell lysate.

The p72 RNA helicase is a member of the DEAD box family of RNA helicases, which are characterized by a conserved motif including Asp-Glu-Ala-Asp (DEAD) and are involved in various biological processes [18,19]. It has been previously reported that p72 directly interacted with ZAP or NZAP-Zeo, and was required for optimal function of ZAP [9]. To examine whether the reduced antiviral activity of the ZAP mutants was caused by failed interaction with the p72 RNA helicase, pull-down assays were performed. Bacterially expressed GST-p72 fusion protein was analyzed for the binding to the ZAP mutants in the presence of RNase A. Nm3, Nm13, Nm103, Nm 113 interacted with p72 as efficiently as the wild-type NZAP-Zeo (Fig. 4). In comparison, the binding of Nm63 and Nm 93 to p72 was reduced, and the binding of Nm133 to p72 was almost diminished (Fig. 4).

## Discussion

ZAP specifically inhibits MLV replication by promoting the degradation of the viral mRNA in the cytoplasm [4]. ZAP directly binds to the viral mRNA and recruits the RNA exosome to degrade the target RNA [7,10]. How ZAP coordinates this process is not clear yet. Here, we used the alanine scanning method to explore the regions important for the antiviral activity of ZAP.

Out of the nineteen mutants tested, seven displayed significantly reduced antiviral activity to both MLV-luc vector and SINV (Fig. 1A and 1C). In an attempt to understand how the activity of these mutants was affected, they were further analyzed for their interaction with the target RNA, the exosome, and the RNA helicase p72, which have been previously reported to be important for the antiviral activity of ZAP [7,9,10]. The results are summarized in table 1.

**Table 1:** Summary of the ZAP mutants for their binding activities to the target RNA, the exosome, and the RNA helicase p72

Mutant	3	13	63	93	103	113	133
**RNA binding**	L	N	L	N	M	N	N
**p72 helicase**	N	N	M	M	N	N	L
**Exosome**	N	N	N	N	N	N	N

N: Normal; M: medium; L: low

The RNA binding activity of Nm3 and Nm 63 was almost depleted, while that of Nm103 was compromised (Fig. 2 and Table 1). These results suggest that the integrity of the overall structure of ZAP may be important for the protein to bind the target RNA and that there may be multiple RNA binding sites. Alternatively, these amino acids may be positioned closely in the tertiary structure such that they form an RNA binding site together. Considering that the minimum length of the ZREs so far identified is about 500 nucleotides [5,10], the former possibility seems more plausible.

All of the seven mutants interacted with the exosome (Fig. 3 and Table 1). However, immunoprecipitation of Rrp46 failed to coprecipitate a truncated ZAP (CZAP429) (Fig. 3), indicating that the interaction between the exosome and the seven ZAP mutants was specific. The specific domain of ZAP required for exosome interaction awaits further identification.

The binding of Nm133 to the RNA helicase p72 was severely impaired, while binding of Nm63 and Nm93 was moderately reduced (Fig. 4 and Table 1). The expression levels of Nm63 and Nm93 were relatively low compared with the other mutants (Fig. 1A). We speculate that these two mutations affected the overall structure of the protein. It is possible that the region around the mutation in Nm133 is the major p72 binding domain, and changes in the protein overall structure affect the binding.

Nm13 and Nm113 bound to the target RNA, the exosome, and p72 normally, suggesting that other mechanisms exist for their reduced antiviral activity. In mammalian cells mRNA degradation is a highly complex process [20,21]. General mRNA degradation starts from deadenylation. The deadenylated mRNA is degraded 3'-5 by the RNA processing exosome. The mRNA is also decapped by the decapping enzyme complex and then degraded 5'-3 by the exoribonuclease XrnI. Co-factors, such as the RNA helicase p72 for ZAP [9], are involved. Furthermore, the activity of trans-acting factor is usually regulated by cellular factors. The properties of Nm13 and Nm113 suggest that other cellular factors may exist that interact with ZAP and are involved in ZAP-mediated RNA degradation.

A mechanism independent of the interaction of ZAP with the target RNA, exosome, or p72 may theoretically also exist. A mutant ZAP that failed to interact with the target RNA, the exosome, or p72, but still retained the antiviral activity would imply the existence of such a mechanism. To explore this possibility, we analyzed the mutants Nm23, 53 and 153, which displayed comparable antiviral activity as wild-type ZAP, for their interaction with the target RNA, the exosome, and p72. These mutants interacted with the target RNA, the exosome, and p72 similarly as the wild-type ZAP (Additional file 1). Further investigation should be needed to explore whether a mechanism exists independent of the interaction of ZAP with the target RNA, exosome or p72.

## Conclusions

We identified seven mutants of ZAP whose antiviral activity was significantly reduced. Five mutants displayed reduced binding to the target RNA or the RNA helicase p72, confirming our previous results. The other two mutants may be useful tools for further understanding the mechanism for ZAP-mediated RNA degradation.

## Methods

### Plasmid construction

The plasmids pBabe-NZAP-Zeo and pNZAP-Zeo-myc have been described previously [4,22]. pBabe-NZAP-Zeo-myc expresses myc-tagged rat NZAP-Zeo. To generate pBabe-NZAP-Zeo-myc, the *Eco*RI-*Cla*I fragment of pBabe-NZAP-Zeo was replaced with *Eco*RI-*Bam*HI and *Bam*HI-*Cla*I PCR-derived fragments. The *Eco*RI-*Bam*HI fragment, which covers the sequence encoding NZAP was PCR-amplified from pNZAP-Zeo-myc using forward primer NZ-SP bearing an *Eco*RI site and reverse primer Bam-AP bearing a silent mutation to create a *Bam*HI site. The *Bam*HI-*Cla*I fragment, which covers the sequence encoding Zeo-myc was PCR amplified from pNZAP-Zeo-myc using forward primer Bam-SP bearing a silent mutation to create a *Bam*HI site, and reverse primer NZ-AP bearing a *Cla*I site.

To generate the alanine substitution mutant, in which three consecutive amino-acids of every ten amino-acids were substituted with three alanines, the *Eco*RI-*Cla*I fragment of pBabe-NZAP-Zeo-myc was replaced with *Eco*RI-*Not*I and *Not*I -*Cla*I PCR-derived fragments. The sequence comprising the *NotI *site and an additional nucleotide encodes three consecutive alanines. The *Eco*RI-*Not*I fragment was PCR amplified from pBabe-NZAP-Zeo-myc using forward primer NZ-SP and reverse primer bearing a *Not*I site. The *Not*I -*Cla*I fragment was PCR amplified from pBabe-NZAP-Zeo-myc using forward primer bearing a *Not*I site and reverse primer NZ-AP. The sequences of the primers are listed below, with the restriction sites in bold.

NZ-SP: 5'-CT**GAATTC**GGCACGAGGCAGCCTCG-3'

Bam-AP: 5'-CG**GGATCC**GCAGGAACGGTCTCTG-3'

Bam-SP: 5'-CG**GGATCC**GCCAAGTTGACCAGTGCC-3'

NZ-AP: 5'-ATATAG**ATCGAT**TCAGCGGGTTTAAACTCA-3'

Nm3-AP: ATATAG**GCGGCCGC**TGCCATGGCGCGCTAT

Nm3-SP: ATATAG**GCGGCCGC**GGTATGCTGTTTCATC

Nm13-AP: ATATAG**GCGGCCGC**CTTGGTGATGAAACAG

Nm13-SP: ATATAG**GCGGCCGC**CGCCCACGGGGGCCGT

Nm23-AP: ATATAG**GCGGCCGC**GGTCATACGGCCCCCG

Nm23-SP: ATATAG**GCGGCCGC**ACTGCTGGGTGAGATC

Nm33-AP: ATATAG**GCGGCCGC**GAGCCTGATCTCACCCA

Nm33-SP: ATATAG**GCGGCCGC**GCAGCTCTACGAGCTG

Nm43-AP: ATATAG**GCGGCCGC**CTCCAGCAGCTCGTAG

Nm43-SP: ATATAG**GCGGCCGC**GCCCGATCGCTTCGTG

Nm53-AP: ATATAG**GCGGCCGC**CAATAGCACGAAGCG

Nm53-SP: ATATAG**GCGGCCGC**AGGCCAGGCCGGGATC

Nm63-AP: ATATAG**GCGGCCGC**CCGAGTGATCCCGGCCT

Nm63-SP: ATATAG**GCGGCCGC**GGCTACTACTCGAGCCCG

Nm73-AP: ATATAG**GCGGCCGC**GACGCGGGCTCGAGTA

Nm73-SP: ATATAG**GCGGCCGC**GAAGTACTGCCAGAGA

Nm83-AP: ATATAG**GCGGCCGC**GCAGGGTCTCTGGCAG

Nm83-SP: ATATAG**GCGGCCGC**GCACCTCTGCAAGCTT

Nm93-AP: ATATAG**GCGGCCGC**CAGATTAAGCTTGCAG

Nm93-SP: ATATAG**GCGGCCGC**GTGCCACTATGCACAG

Nm103-AP: ATATAG**GCGGCCGC**CTGAGACTGTGCATAG

Nm103-SP: ATATAG**GCGGCCGC**CTGCAAATATTCTCAC

Nm113-AP: ATATAG**GCGGCCGC**AACATCGTGAGAATA

Nm113-SP: ATATAG**GCGGCCGC**ACAGAACTTCCAGAT

Nm123-AP: ATATAG**GCGGCCGC**CTTCAGGATCTGGAAG

Nm123-SP: ATATAG**GCGGCCGC**GCTCTCTGGGCTTAAC

Nm133-AP: ATATAG**GCGGCCGC**CTCTTGGTTAAGCCCA

Nm133-SP: ATATAG**GCGGCCGC**TTGCCTCCTGGTCCAAAG

Nm143-AP: ATATAG**GCGGCCGC**GTCGCTTTGGACCAGGA

Nm143-SP: ATATAG**GCGGCCGC**CCTGCCCGAGATATGC

Nm153-AP: ATATAG**GCGGCCGC**ACTCTTGCATATCTC

Nm153-SP: ATATAG**GCGGCCGC**AGAGGGCCGAAAACAG

Nm163-AP: ATATAG**GCGGCCGC**ACAGGTCTGTTTTCGG

Nm163-SP: ATATAG**GCGGCCGC**ACAGCCATGCGAGAGA

Nm173-AP: ATATAG**GCGGCCGC**GTGGAGTCTCTCGCAT

Nm173-SP: ATATAG**GCGGCCGC**GCACTTCACCCGGGGC

Nm183-AP: ATATAG**GCGGCCGC**GCAGTTGCCCCGGGTG

Nm183-SP: ATATAG**GCGGCCGC**CAACTGTCTCAGGTCT

Nm193-AP: ATATAG**GCGGCCGC**GTTGTGAGACCTGAGAC

Nm193-SP: ATATAG**GCGGCCGC**CAGAAAGGTGTTGACCA

Nm203-AP: ATATAG**GCGGCCGC**CATGATGGTCAACACC

Nm203-SP: ATATAG**GCGGCCGC**CGGGCTGAGTCCTGAT

Nm213-AP: ATATAG**GCGGCCGC**GACCACATCAGGACTC

Nm213-SP: ATATAG**GCGGCCGC**CCAGGACATCTGCAAC

Nm223-AP: ATATAG**GCGGCCGC**TTTGTTGTTGCAGATG

Nm223-SP: ATATAG**GCGGCCGC**GAGGAACCCGCCTGGC

Nm233-AP: ATATAG**GCGGCCGC**TCTCGTGCCAGGCGGGT

Nm233-SP: ATATAG**GCGGCCGC**TCCACACCGCAGAGGC

Nm243-AP: ATATAG**GCGGCCGC**TGCGCCGCCTCTGCGGT

Nm243-SP: ATATAG**GCGGCCGC**CAGAAGCAAAAGCAGA

pcDNA4/TO/myc-ZAP was previously described as pZAP-myc [4]. Zm3, Zm13, Zm23, Zm53, Zm63, Zm93, Zm103, Zm113, Zm133 and Zm153 express myc-tagged full-length ZAP containing the alanine substitutions corresponding to those in Nm3, Nm13, Nm23, Nm53, Nm63, Nm93, Nm103, Nm113, Nm133 and Nm153, respectively. To generate Zm3, the PCR fragment amplified with Z-SP/Mid-AP as primers and Nm3 as template was digested with *Bam*HI and *Nhe*I and used to replace the *Bam*HI-*Nhe*I fragment of pcDNA4/TO/myc-ZAP. The same strategy was employed to generate Zm13, Zm23, Zm53, Zm63, Zm93, Zm103 and Zm113. To generate Zm133, the PCR fragment generated using Nm133 as template and Z-SP/Mid-RP as primers, and the PCR fragment using pcDNA4/TO/myc-ZAP as template and Mid-SP/Z-AP as primers were mixed and amplified using PCR primers Z-SP and Z-AP. The resulting *Bam*HI-*Eco*RI fragment was used to replace the *Bam*HI-*Eco*RI fragment of pcDNA4/TO/myc-ZAP. The same strategy was employed to generate Zm153.

Z-SP: CT**GGATCC**GGCACGAGGCAGCCTCG

Mid-AP: TCTGTGTGCGCCGCCTCTGCGGTGT

Mid-SP: ACACCGCAGAGGCGGCGCACACAGA

Z-AP: TTTGCCTG**GAATTC**CTGAGACCGAT

pcDNA4/TO/myc-CZAP429 expresses myc-tagged CZAP429(amino acids 429-776 of ZAP). To generate pcDNA4/TO/myc-CZAP429, a ZAP fragment was amplified by using forward primer CZAP429SP bearing a *Bam*HI site and reverse primer CZAP429AP bearing a *Not*I site and was used to replace the *Bam*HI-*Not*I fragment of pcDNA4TO/myc-ZAP.

CZAP429SP: CTGGATCCATGGCACAGGATCTGCAGACCACA

CZAP429AP: ACTCGAGCGGCCGCCCTCTGGACCTCTTC

### Cell Culture

All the cells were maintained in DMEM supplemented with10% FBS. Transfection was performed using Fugene 6 (Roche Diagnostics) according to the manufacturer's instruction. Rat2-HA-Zeo and Rat2-NZAP-Zeo cells have been described previously [4]. The pBabe-NZAP-Zeo-myc based constructs expressing NZAP-Zeo-myc mutants were packaged into MLV vector to transduce Rat2 cells. The cells were selected with zeocin (100 μg/ml), and zeocin-resistant cells were pooled for further analyses.

MLV-luc has been previously reported [4]. To evaluate the antiviral activities of the NZAP-Zeo-myc mutants, cells were seeded in 35 mm dishes and infected with MLV-luc on the next day. Infection was conducted for 3 h followed by replacement of the infection medium with fresh medium. 48 hours later, the cells were lysed and luciferase activities were measured. Fold inhibition was calculated as the luciferase activity in the Rat2-HA-Zeo control cells divided by the luciferase activity in the cells expressing the NZAP-Zeo-myc mutants.

The methods for SINV infection and titration have been previously described [10]. Briefly, cells were seeded at 7 × 10^5 ^in six-well dishes the day prior to infection. The next day, the cells were infected with the Toto1101 virus (MOI of 1) for 1 h. The titer of the stock was determined on BHK21 cells. After infection, the cells were washed twice with medium, and 2 ml of fresh medium was added. At 48 h post infection, the supernatants were collected and titrated in duplicate wells using permissive BHK21 cells.

293TRex and 293TRex-ZAP cell lines have been described previously [10]. To generate 293TRex cell lines expressing the ZAP mutants in a tetracycline-inducible manner, pcDNA4/TO/myc-ZAP mutants were stably transfected into 293TRex cells and selected in zeocin-containing medium. Zeocin resistant cells were pooled and used for further analyses.

### In vitro RNA binding assay

The method has been described previously [10].

### Co-immunoprecipitation

Cells were lysed in lysis buffer B (30 mM Hepes pH7.6, 100 mM NaCl, 0.5% NP-40 and protease inhibitors cocktail) on ice for 10 minutes, and the lysates were clarified by centrifugation at 4°C for 10 minutes at 13000 rpm. The supernatant was mixed with proteinG plus-agarose (Santa Cruz Biotechnology) and the antibody, and incubated at 4°C for 2 h. The resins were then washed 3 times with lysis buffer B, and the bound proteins were detected by Western blotting.

### Pull down assay

GST fusion proteins were immobilized on glutathione Sepharose 4B and then incubated with the lysate of the cells expressing the NZAP-Zeo-myc mutants in the presence of RNase A (100 μg/ml) for 2 h at 4°C. The resin was washed three times with PBS, and then analyzed by SDS-PAGE and Western blotting.

## Competing interests

The authors declare that they have no competing interests.

## Authors' contributions

Author contributions: GG, FL and XW designed research; XW and FL performed research; XW, FL and GG analyzed data; and GG drafted the manuscript. All authors read and approved the final manuscript.

## Supplementary Material

Additional file 1 **ZAP mutants 23, 53 and 153 interacted with the target RNA, the exosome and the p72 RNA helicase similarly as the wild-type ZAP**. Nm 23, 53 and 153 were assayed for their interaction with the target RNA (A), the exosome (B) and the RNA helicase p72 (C) as described in the legends to Figure 2, 3 and 4, respectively. Click here for file
