# Supplementary material for: Mutagenesis analysis of the zinc-finger antiviral protein
Source: Retrovirology. 2010 Mar 13;7:19. doi: 10.1186/1742-4690-7-19 (PMC2847535; doi:10.1186/1742-4690-7-19)
Supplement: Additional file 1 — ZAP mutants 23, 53 and 153 interacted with the target RNA, the exosome and the p72 RNA helicase similarly as the wild-type ZAP. Nm 23, 53 and 153 were assayed for their interaction with the target RNA (A), the exosome (B) and the RNA helicase p72 (C) as described in the legends to Figure 2, 3 and 4, respectively. [file 1742-4690-7-19-S1.PDF]

Additional File

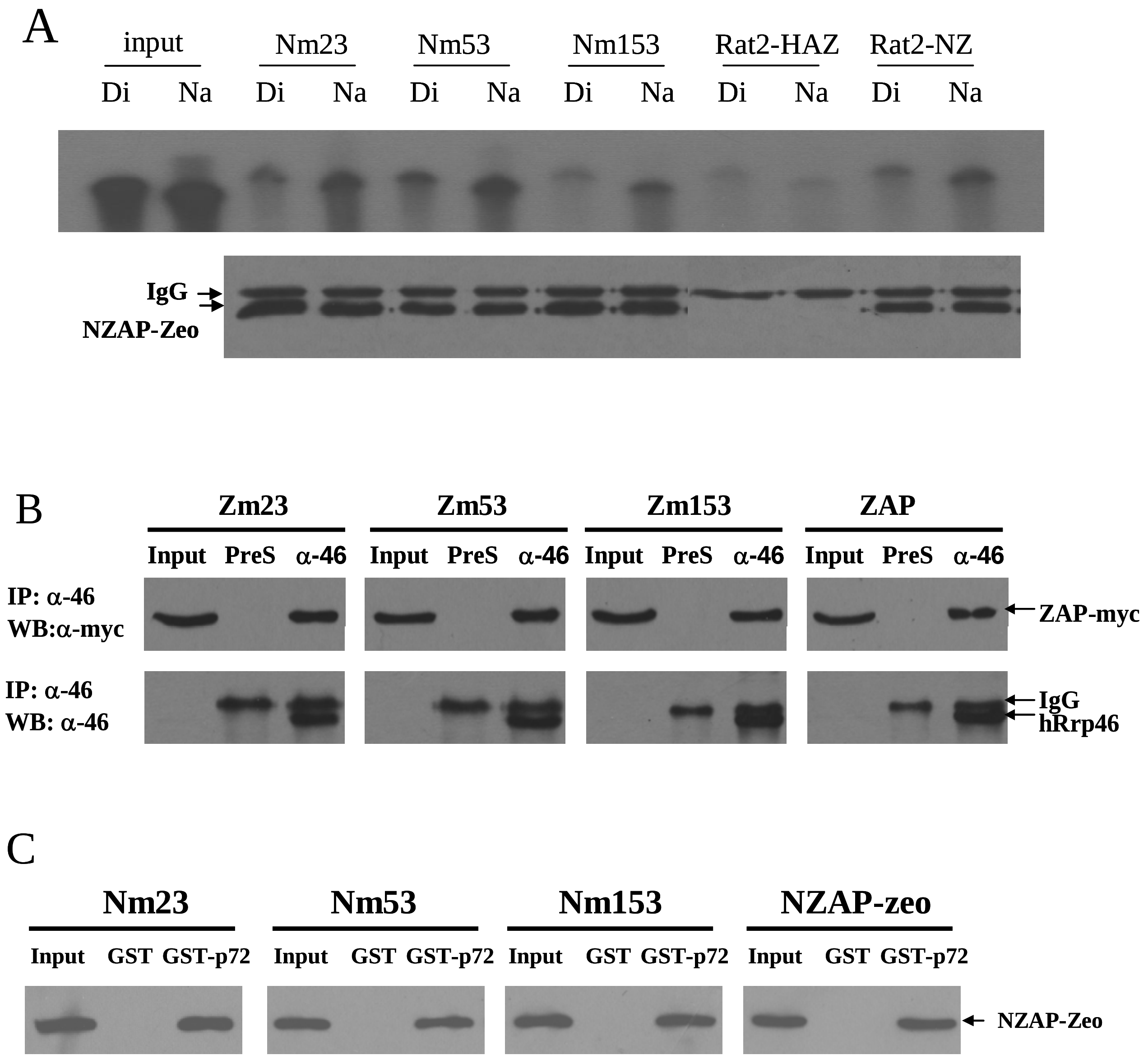

ZAP mutants 23, 53 and 153 interacted with the target RNA, the exosome and the p72 RNA helicase similarly as the wild-type ZAP. Nm 23, 53 and 153 were assayed for their interaction with the target RNA (A), the exosome (B) and the RNA helicase p72 (C) as described in the legends to Figure 2, 3 and 4, respectively.
